# Barley disease susceptibility factor RACB acts in epidermal cell polarity and positioning of the nucleus

**DOI:** 10.1093/jxb/erw141

**Published:** 2016-04-07

**Authors:** Björn Scheler, Vera Schnepf, Carolina Galgenmüller, Stefanie Ranf, Ralph Hückelhoven

**Affiliations:** Phytopathology, Technische Universität München, D-85354 Freising-Weihenstephan, Germany

**Keywords:** *Blumeria graminis*, disease susceptibility, epidermis, MAP kinase, nucleus, oxidative burst, polarity, ROP GTPase.

## Abstract

RHO GTPases are regulators of cell polarity and immunity in eukaryotes. In plants, RHO-like RAC/ROP GTPases are regulators of cell shaping, hormone responses, and responses to microbial pathogens. The barley (*Hordeum vulgare* L.) RAC/ROP protein RACB is required for full susceptibility to penetration by *Blumeria graminis* f.sp. *hordei* (*Bgh*), the barley powdery mildew fungus. Disease susceptibility factors often control host immune responses. Here we show that RACB does not interfere with early microbe-associated molecular pattern-triggered immune responses such as the oxidative burst or activation of mitogen-activated protein kinases. RACB also supports rather than restricts expression of defence-related genes in barley. Instead, silencing of RACB expression by RNAi leads to defects in cell polarity. In particular, initiation and maintenance of root hair growth and development of stomatal subsidiary cells by asymmetric cell division is affected by silencing expression of RACB. Nucleus migration is a common factor of developmental cell polarity and cell-autonomous interaction with *Bgh*. RACB is required for positioning of the nucleus near the site of attack from *Bgh*. We therefore suggest that *Bgh* profits from RACB’s function in cell polarity rather than from immunity-regulating functions of RACB.

## Highlight

The barley GTPase RACB is a disease susceptibility factor. However, RACB is not a key regulator of plant immune responses but acts in polar cell development.

## Introduction

Plants possess an innate immunity, which constantly monitors the cell surface and cytoplasm for the presence or activity of pathogenic organisms. Plant immune receptors can detect conserved molecular patterns that derive from microbes (MAMPs, microbe-associated molecular patterns) or from host cell damage (damage-associated molecular patterns). Such receptors are called pattern recognition receptors (PRRs). They are localized in the host plasma membrane and function in basal resistance to non-adapted and virulent pathogens (Macho and Zipfel, 2014). Additionally, a second class of plant immune receptors co-evolved with specific, largely polymorphic virulence effectors (Jones and Dangl, 2006). Most of these so-called resistance (R) proteins are localized in the cytoplasm and nucleoplasm. For triggering immunity, R proteins directly interact with effector proteins, monitor functionality of effector targets, or mimic effector targets (Dodds and Rathjen, 2010). Plant immunity is robust in most environments. Nevertheless, microbes adapt to plant hosts by evolution of virulence effectors that suppress or circumvent host immunity.

Plant disease resistance can be observed as a result of MAMP- or effector-triggered immunity but also as a consequence of mutations of susceptibility genes. Susceptibility genes encode host factors that are required for pathogenesis in interactions of susceptible hosts with adapted virulent pathogens. Mechanistically, loss of susceptibility can result from de-regulated or primed immunity if susceptibility genes code for negative regulators of plant defence. Alternatively, loss of susceptibility can be explained by lack of or mutation of effector targets that serve demands of the pathogen other than suppressing immunity. Furthermore, susceptibility factors might provide developmental or metabolic prerequisites for attraction, accommodation, or feeding of the pathogen (Hückelhoven *et al.*, 2013; Lapin and Van den Ackerveken, 2013). Loss of susceptibility is usually recessively inherited after loss of gene function and often accompanied by pleiotropic effects that limit applicability in plant breeding (Pavan *et al.*, 2010; Hückelhoven *et al.*, 2013; van Schie and Takken, 2014). Thus a deeper understanding of susceptibility is required to inform plant breeding.

Plant monomeric RHO GTPases (rat sarcoma homologues, also called RAC for rat sarcoma-related C3 botulinum toxin substrate or ROP for RHO of plants) are involved in immunity and susceptibility to plant diseases. Type I RAC/ROPs possess a typical motif for post-translational prenylation at their C-terminus, and can be additionally palmitoylated after activation. In contrast, type II RAC/ROPs are often constitutively *S*-acylated (Yalovsky, 2015). In rice, the type II RAC/ROP protein RAC1 is a central regulator of immune response mediated by either PRRs or R proteins (Kawano *et al.*, 2014). The rice chitin elicitor PRR CERK1 can activate RAC1 via a plant-specific guanine nucleotide exchange factor RACGEF1. RAC1 orchestrates the elicitor-activated production of reactive oxygen species (ROS), mitogen-activated protein (MAP) kinase (MAPK) activation, transcriptional responses, and changes of the host proteome (Kawano *et al.*, 2014). Additionally, RAC1 is activated downstream of the R protein Pit, a nucleotide-binding leucine-rich repeat protein, which confers effector-triggered immunity to the rice blast fungus *Magnaporthe oryzae* (Kawano *et al.*, 2010). However, in rice and barley, there are also other type I and type II RAC/ROP GTPases that limit basal resistance or support susceptibility to fungal diseases (Chen *et al.*, 2010; Schultheiss *et al.*, 2002, 2003). Barley RACB, a type I RAC/ROP, is required for full susceptibility to barley powdery mildew caused by the biotrophic ascomycete *Blumeria graminis* f.sp. *hordei* (*Bgh*). Transient or stable gene silencing of *RACB* by RNAi limits fungal entry and formation of fungal haustoria in barley epidermal cells (Schultheiss *et al.*, 2002; Hoefle *et al.*, 2011). Molecular cell biology suggests a role for RACB in organization of the cytoskeleton (Opalski *et al.*, 2005; Hoefle *et al.*, 2011; Huesmann *et al.*, 2012). Transgenic expression of constitutively activated (CA) RACB (*RACB* ^*G15V*^) enhances powdery mildew susceptibility, supports establishment of haustoria in barley epidermal cells, but has little effect on cellular defence reactions. When overexpressed in single epidermal cells or transgenic plants, barley type II CA RAC/ROPs (RAC1, RAC3, and ROP6) can support susceptibility to powdery mildew too, whereas RACD, another type I RAC/ROP, appears not to influence the outcome of interaction with *Bgh* (Schultheiss *et al.*, 2003; Pathuri *et al.*, 2008). Little is known about the function of barley RAC/ROPs in interaction with other microbes. However, when ectopically expressed, barley CA RACB and CA RAC3 can support susceptibility to *Pseudomonas syringae* pv. *tabaci* in tobacco, and barley CA RAC1 can support penetration resistance to hemibiotrophic *M. oryzae* in transgenic barley (Pathuri *et al.*, 2008, 2009).

RAC/ROPs function in plant cell polarity. This is well established for root hair tip growth, pollen tube tip growth, and epidermal pavement cell interdigitation (Yang, 2008). In particular, type I RAC/ROP GTPases of dicots function in cell polarity. Little is known about RAC/ROP functions in polar cell growth in monocots. It has been described that barley *CA RACB* enhances epidermal cell size in leaves. Root hair phenotypes of transgenic *CA RACB* barley were reported as root hair swelling on solid medium, which is typical in dicots expressing *CA ROP* genes (Jones *et al.*, 2002; Pathuri *et al.*, 2008, 2009). In contrast, silencing of *RACB* by RNAi in transgenic barley led to a defect in the ability to form root hairs (Hoefle *et al.*, 2011). In maize, the development of stomatal complexes was reported to depend partially on ROP2 and ROP9, two type I RAC/ROP proteins very similar to barley RACB (Humphries *et al.*, 2011).

It has not been studied whether RACB interferes with pattern-triggered immunity or defence gene expression in response to *Bgh*. Here, we show that RACB does not limit early MAMP-triggered immune responses and supports rather than limits expression of defence genes. However, knock down of *RACB* strongly affects polar cell growth and positioning of the nucleus in barley epidermal cells. *Bgh* may hence profit from functions of RACB in cell polarity during invasion of host cells.

## Materials and methods

### Plant material and growth conditions

For all experiments, the barley (*Hordeum vulgare*) cultivar Golden Promise and transgenic *RACB* plants with the genetic background of Golden Promise were used. The overexpressor line of CA RACB 17/1-11 and RACB RNAi 16/2-4B and 15/1-16 have been described previously (Schultheiss *et al.*, 2005; Hoefle *et al.*, 2011). Kernels were surface-sterilized in 20ml of sterilization solution (4% NaOCl, Tween-20) for 1.5h with shaking. After washing with H_2_O for 30min, husks were carefully removed without damaging the embryo to guarantee equal germination of seeds. Seeds were pre-germinated on wet filter paper for 2 d in the dark before being sown into soil (Typ ED73, Einheitserde- und Humuswerke, Gebr. Patzer GmbH & Co KG, Sinntal-Jossa, Germany). Plants were grown in a growth chamber (Conviron, Winnipeg, Canada) at 18 °C with relative humidity of 65% and a photoperiod of 16h. Both transgenic genotypes do not produce homozygous offspring. Offspring of transgenic T_3_ donor plants were genotyped according to previous studies to separate transgenic offspring carrying the T-DNA from azygous offspring that lost the T-DNA due to segregation. Azygous sister plants are similar to the wild type (WT; Schultheiss *et al.*, 2005; Hoefle *et al.*, 2011) and thus served as ideal controls. *Arabidopsis thaliana* ecotype Columbia 0 (Col-0) seeds were purchased from Lehle Seeds (Round Rock, USA) and stratified for 2^d at 4 °C before placing into a growth chamber. Plants were grown at 22 °C with a photoperiod of 10h and a relative humidity of 65%.

### Elicitors

The flagellin elicitor flg22 (Felix *et al.*, 1999) was synthesized as described before (Ranf *et al.*, 2011). Chitin from shrimp shells (Sigma-Aldrich Chemie GmbH, Steinheim, Germany) was ground to a fine powder and suspended in H_2_O (20mg ml^–1^). Insoluble chitin fragments were removed by centrifugation (1900 *g*, 10min) and the supernatant was used for experiments.

### Immunoblot analysis

For detection of activated MAPKs, we used 10 leaf discs of 5mm diameter from second leaves of 14-day-old barley plants or from 6-week-old Arabidopsis plants per time. Leaf discs were incubated in 2ml of H_2_O/well for 16h in 24-well plates, transferred to fresh H_2_O for 30min, and subsequently elicited with 1 µM flg22 or 100 µg ml^–1^ chitin. Detection of activated MAPKs with anti-pTEpY (α-phospho-p44/42-ERK, Cell Signaling Technology, Boston, USA) was performed as previously described (Saijo *et al.*, 2009; Ranf *et al.*, 2011).

### Detection of ROS production of barley leaves

ROS production was assayed by H_2_O_2_-mediated oxidation and luminescence of the luminol derivative L-012 (Wako Chemicals GmbH, Neuss, Germany). Leaf discs (5mm diameter) of 7-day-old barley plants were floated in 200 µl of H_2_O/well overnight in a 96-well plate. After removal of H_2_O, leaf discs were incubated for 30min in 2 µg ml^–1^ horseradish peroxidase (HRP) and 10 µM L012. Subsequently, leaf discs were elicited with 100nM flg22 or 100 µg ml^–1^ chitin. Luminescence was measured at 1min intervals with a Tecan Reader (infinite M200, Tecan, Männedorf, Switzerland) for 30min. We calculated relative luminescence units (RLU) by subtraction of leaf disc-specific background (recorded for 5min before elicitation) and of mock treatment-associated blanks.

### Quantitative reverse transcription PCR

Gene expression analysis was carried out by reverse transcription quantitative real-time PCR (RT–qPCR) in a Mx3005P cycler (Agilent Technologies, Santa Clara, CA, USA) using the Maxima SYBR Green qPCR master mix (2×) (Thermo Fisher Scientific, St. Leon-Rot, Germany). Reactions were performed in duplicate with 10ng of cDNA and 330nM forward and reverse primer each in a final volume of 10 µl. Expression values of defence genes and *RACB* were normalized to a barley housekeeping ubiquitin (*HvUBI*) (Ovesna *et al.*, 2012) using primer efficiency correction as suggested by Pfaffl (2001). The program consisted of an initial step at 95 °C for 10min and 95 °C for 30s, followed by 40 cycles at 55 °C for 30s and at 72 °C for 1min. The melting curve analysis was performed at 55–95 °C. All primers (Table 1) were designed using Primer3 software (Untergasser *et al.*, 2012) and were checked for specificity using the Basic Local Alignment Search Tool (BLAST) and therein with nucleotide blast against the *H. vulgare* database (http://blast.ncbi.nlm.nih.gov), and amplicon size assessment in agarose gels before running RT–qPCR.

**Table 1.** Oligonucleotides for RT–qPCR

Gene	Accession number	Forward primer (5'→3')/reverse primer (5'→3')	Annealing temperature (°C)	Product size (bp)
*HvUBI*	AK252410, M60175	TCTCGTCCCTGAGATTGCCCACAT/TTTCTCGGGACAGCAACACAATCTTCT	58	263
*HvPR1*	Z26333	AAGCTGCAAGCGTTCGCC/AGGTGTTGGAGCCGTAGTC	60	184
*HvPR3*	AK364132	CTACACGTACGACGCCTTCAT/GTGGCCTTGCTTATCTCTTCC	60	194
*HvPR5*	AK371265 AJ001268	CACGGACATCACCAAGGATT/TTGCCCTTGAAGAACATTGAG	60	152
*HvPR10*	AK360974	AGGGCGACAAGGTAAGTGG/CATCTTGAGCAGGTCGAGGTA	60	181
*HvJIP23*	AB251339	TGTTGCAGACTATGCCATGAA/TGCCAATCGTTGTACTTAGCC	60	167
*HvJIP60*	AK372562	TTCTTCTTCCGGGCTGTAAAT/GTACGCTGAGCTACCCAGACA	60	150
*HvRACB*	AJ344223	TGCACCAGGTGTGCCTATTATC/CTTCGCCCTTGTTCTTTGTC	60	309

### Scanning electron microscopy

For scanning electron microscopy (SEM), root and leaf material was harvested and fixed in 4% paraformaldehyde (4% PFA) in 1× phosphate-buffered saline buffer (1× PBS), pH 7.4 as described (Sauer and Friml, 2010). Fixed material was washed in 1× PBS, pH 7.4 three times for 10min, followed by three washing steps in distilled water for 10min. Dehydration occurred in an increasing ethanol series of 25% (v/v), 50% (v/v), and 75% (v/v) in distilled water and pure ethanol three times each for at least 10min. For critical point drying, the EM CPD300 Automated Critical Point Dryer (Leica, Vienna, Austria) was used and drying was done following the ‘Rice Root’ protocol for root tissue and the ‘Tobacco Leaf’ protocol for barley leaf material as described in the manufacturer′s manual. The imaging was done using the TM300 Tabletop Microscope (Hitachi, Tokyo, Japan). The image editing program GIMP 2.8 was used to merge single root pictures to generate root overviews and to colour subsidiary cells in the leaf images.

### Fluorescence microscopy of root tissue

Seedling roots were harvested, fixed, and washed as described above. For staining, propidium iodide (PI; Applichem, Darmstadt, Germany) was dissolved in distilled water to a final concentration of 100 µg ml^–1^ for RACB RNAi root material and 40 µg ml^–1^ for the azygous control plants. The root material was incubated in the staining solution for 1h in the dark. Subsequently, stained roots were transferred to a clearing solution, prepared by mixing chloral hydrate, glycerol, and water in the ratio 4:1:2 (w/v/v) and kept there for 15h. After clearing, the roots were directly mounted in Hoyer’s solution consisting of 1g of glycerol, 10g of chloral hydrate, and 1.5g of gum arabic dissolved in 2.5ml of distilled water. Visualization followed immediately using a Leica TCS SP5 Confocal Microscope and the Leica LAS AF software (Leica Microsystems, Mannheim, Germany). PI was excited by a 561nm laser line and emission was detected from 560nm to 675nm.

### Measurement of the nucleus attraction index

At 8h after inoculation, the leaf material was harvested and halved along the longitudinal axis using a razor blade. One half of the leaf blade was used for relative quantification of RACB expression. Leaf pieces were fixed, de-waxed, and destained (Sauer and Friml, 2010). For the last rehydration step, 1× PBS, pH 7.4 was used. To remove RNA from the tissue, RNase A (DNase free, Applichem, Darmstadt, Germany) was dissolved in 10mM Tris–HCl, pH 7.5 to a final concentration of 10mg ml^–1^. The stock solution was subsequently diluted in 1× PBS, pH 7.4 to 100 µg ml^–1^ to achieve the RNase A solution in which leaf material was incubated for 1h for RNA digestion. Subsequently the leaves were placed in the staining solution (100 µg ml^–1^ PI in distilled water) for at least 5min. To determine the nucleus attraction index (NAI), epidermal B cells (Koga *et al.*, 1990), which were attacked by a single fungal appressorium, were imaged. A *z*-stack was recorded starting from the brightest fluorescence of the fungal appressorium to the brightest fluorescence of the plant nucleus. The picture number and increments were adjusted for each cell and *z*-stack, depending on the vertical distance between the appressorium and plant nucleus. The NAI was calculated as follows: $NAI=\;\sqrt{a^{2}+b^{2}}/d,$ where *a* reflects the depth of the *z*-stack and *b* the planar distance between the appressorium and the nucleus. Both represent the legs of a right-angled triangle. The diagonal of the B cell is represented by *d*. Cell size measurement was performed using the software ImageJ (Schneider *et al.*, 2012).

### Rhodamine 123 staining and trichoblast quantification

Rhodamine 123 (R123) selectively stains mitochondria in living cells (Wu, 1987). A stock solution was prepared by dissolving R123 (Sigma-Aldrich, St Louis, MO, USA) in DMSO to a final concentration of 10mg ml^–1^ For the staining solution, the stock solution was diluted in 0.5× Murashige and Skoog medium with modified vitamins (0.5× MS; Duchefa Biochemie, Harleem, The Netherlands) mixed with sucrose to 1% (w/v) and 2-(*N*-morpholino)ethanesulphonic acid (MES; Carl Roth, Karlsruhe, Germany) to 0.05% (w/v) final concentration, pH 5.6 to 1 µg ml^–1^. Intact seedlings were incubated in the staining solution for 10min in the dark. After staining, seedlings were briefly rinsed in an excess of 0.5× MS, pH 5.6 and immediately visualized by confocal microscopy. R123 was excited by a 488nm laser line and the emission was detected from 515nm to 575nm. In an early developmental state, trichoblasts were counted before root hair initiation each in an area of 0.024mm^2^.

## Results

### RACB does not control early MAMP responses in barley

Specific RAC/ROP GTPases modulate immune responses and NADPH oxidase-dependent ROS production in plants. Therefore, we tested barley WT Golden Promise and corresponding transgenic barley plants silenced for *RACB* by RNAi or overexpressing *CA RACB* for their ability to respond to MAMPs by production of ROS. Transgenic lines used have been validated before as being silenced or overexpressors, respectively, and are representatives of several (*CA RACB*) or two (*RACB* RNAi) independent lines with consistent transgene-associated phenotypes (Schultheiss *et al.*, 2005; Hoefle *et al.*, 2011). The barley type I RAC/ROP gene *RACD* is co-silenced in the *RACB* RNAi line, whereas other RAC/ROPs show WT-like expression (Hoefle *et al.*, 2011).

**Fig. 1.**
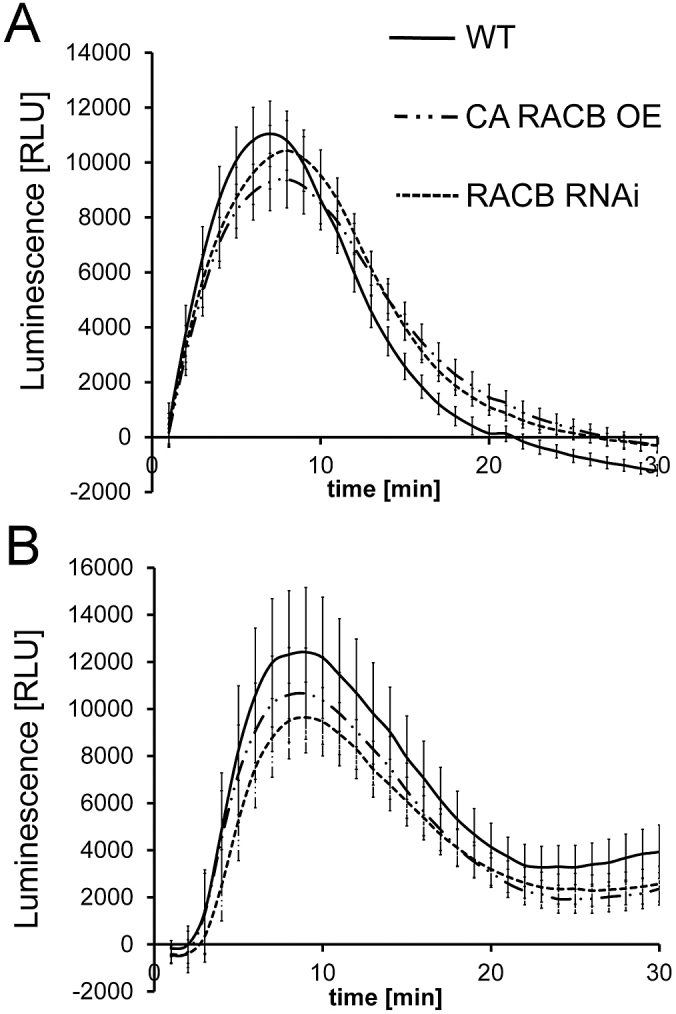
MAMP-triggered ROS burst is unaffected by *RACB* transgenes. (A) Chitin- (100 µg ml^−1^) triggered ROS in barley is unaffected by overexpression of *CA RACB* (RACB OE) or by silencing *RACB* (*RACB* RNAi). (B) Flagellin- (100nM flg22) triggered ROS in barley is unaffected by *CA RACB* (RACB OE) or by silencing *RACB* (*RACB* RNAi). Elicitors were added to leaf discs at 0min and ROS-dependent luminol luminescence recorded over 30min. Data show relative luminescence units (RLU) that have been corrected by subtraction of leaf disc-specific background (recorded for 5min before elicitation) and mock treatment-associated blanks (average of eight leaf discs). Error bars show the SE over the mean of four (A) or three (B) experiments each with eight elicited leaf discs per genotype.

WT barley plants showed a typical MAMP-triggered oxidative burst when challenged with a chitin elicitor preparation. No ROS burst was recordable in mock-treated plants. After elicitor treatment, barley leaf discs rapidly produced ROS. The kinetics and amount of ROS produced appeared similar in WT, *CA RACB*, and *RACB* RNAi barley (Fig. 1A). To test whether RACB might influence the oxidative burst elicited by a fungus-unrelated MAMP, we included the bacterial flagellin-derived elicitor flg22 in our experiments (Felix *et al.*, 1999). The flg22 peptide elicited an oxidative burst that was, when taking variance of biological repetitions into account, indistinguishable between WT, *CA RACB*, and *RACB*-RNAi barley (Fig. 1B).

**Fig. 2.**
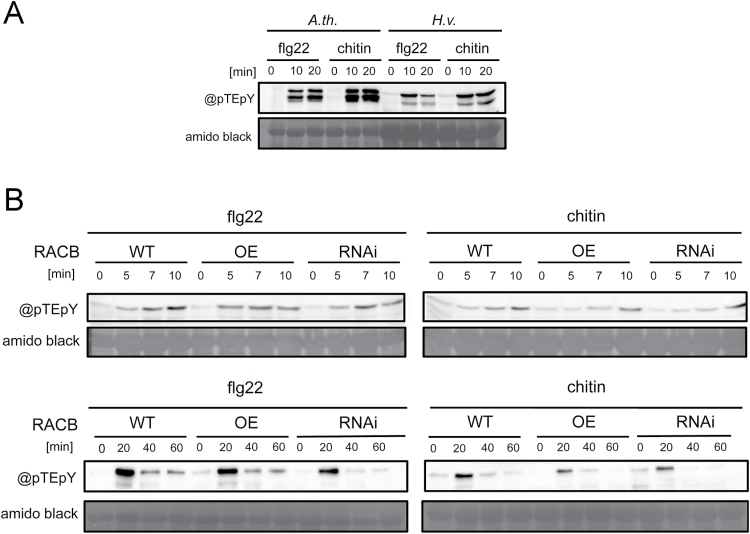
MAMP-triggered MAPK phosphorylation is unaffected by *RACB* transgenes. (A) Elicitation of Arabidopsis (A.th.) or barley (H.v.) leaf discs induced phosphorylation of similar MAPKs as detected by an antibody (@pTEpY) that detects the phosphorylated TEY motif in MAPKs (Ranf *et al.*, 2011) (B) Chitin- (100 µg ml^−1^) or flagellin- (100nM flg22) triggered MAPK phosphorylation in barley is unaffected by overexpression of *CA RACB* (OE) or by silencing *RACB* (RNAi). Elicitors were added to leaf discs at 0min and proteins extracted at 5, 7, and 10min or at 20, 40, and 60min.

MAPK activation is another typical early MAMP response and potentially modified by plant RAC/ROPs. Activation of MAPKs can be detected by immunodetection of a phosphorylated MAPK-typical TEY motif (pTEpY), such as that present in Arabidopsis MPK3 and MKP6 (Ranf *et al.*, 2011). We detected phosphorylated MAPKs (MAPK-P) in Arabidopsis and barley leaf discs treated in parallel either with chitin or with flg22 (Fig. 2A). In Arabidopsis, typical phosphorylation of MAPKs was detected after elicitation with chitin or flg22. Both elicitors induced a similar pattern of activated MAPK-P in protein extracts from barley leaf discs. However, one or two bands appeared predominant in most experiments, whereas in Arabidopsis extracts two to three bands were detected. This suggested that barley reacts to MAMPs with activation of MAPKs. We then compared patterns of MAPK-P in WT barley, *CA RACB* barley, and *RACB* RNAi barley (Fig. 2B). This revealed, at the given level of detection, similar MAPK activation in all three genotypes, with a slight increase visible after 5min and a decline between 20min and 40min after elicitation. This suggests that activity and abundance of RACB do not strongly influence barley competence to react to MAMPs with typical early MAMP responses.

**Fig. 3.**
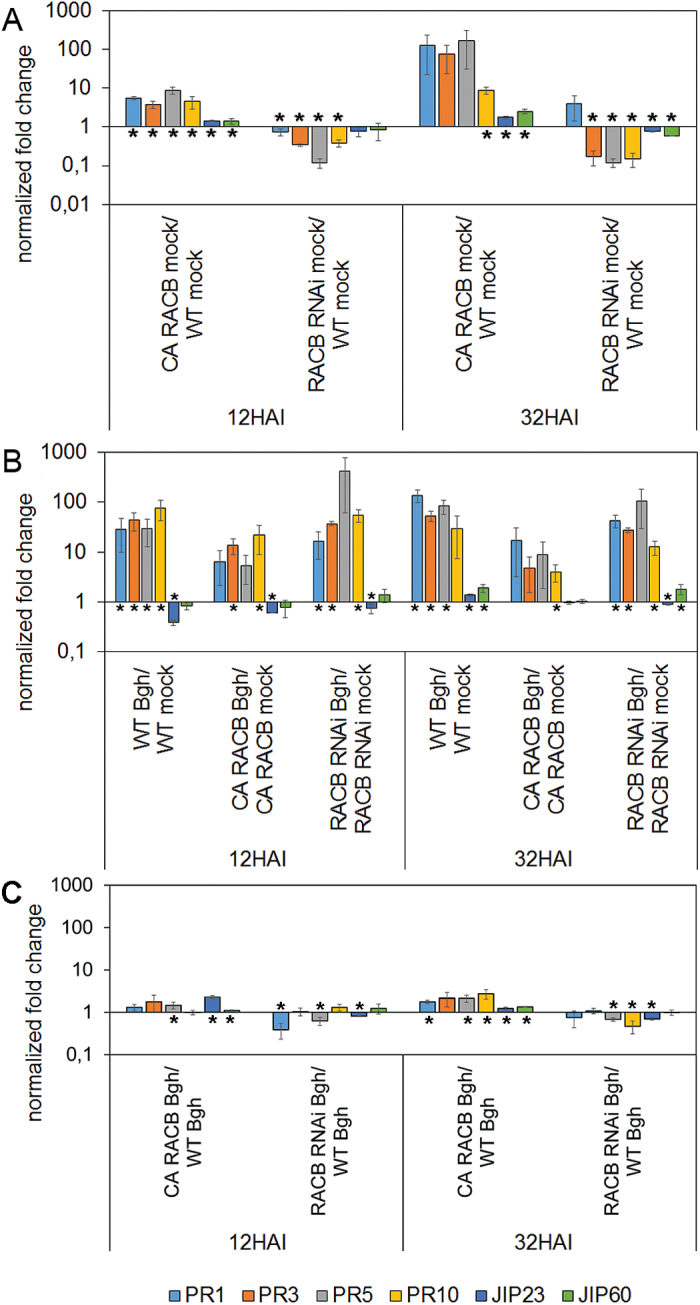
Marker gene expression does not reflect the susceptibility status of *RACB*-transgenic barley. Wild-type (WT), *CA RACB*-overexpressing, and *RACB* RNAi plants were either mock-treated or inoculated with *Bgh* (100 spores mm^−2^) and collected at 12 or 32 HAI for RT–qPCR (A) Genotype-dependent expression of defence-related genes [pathogenesis-related (*PR*) genes and jasmonate-induced protein (JIP) genes]. Genes are constitutively overexpressed in mock-treated *CA RACB* plants versus WT plants and partially underexpressed in *RACB* RNAi plants. (B) *Bgh*-triggered expression of *PR* genes and *JIP* genes is only weakly affected by the transgenes. Less strong PR gene expression in *CA RACB* plants is explained because genes are already constitutively expressed on a higher level (see A). (C) Genotype-dependent expression of *PR* genes and JIP genes in *Bgh*-inoculated plants. Columns show the average fold change of three biological repetitions of gene expression relative to that of constitutively expressed *HvUBI*. Error bars show the SE of three fully independent experiments. * indicate significant changes at *P*<0.05 according to a two-sided one-sample *t*-test.

We further studied pathogenesis-related (*PR*) *PR1*, *PR3*, *PR5*, and *PR10* gene expression after high density inoculation with *Bgh*, because the strength of *PR* gene expression has been linked to penetration resistance in barley (Peterhänsel *et al.*, 1997; Molitor *et al.*, 2011). We further wanted to test whether RACB possibly acts as a negative regulator of *PR* or jasmonate-associated gene expression (jasmonate-induced genes *JIP23* and *JIP60*) (Kogel *et al.*, 1995). We compared gene expression after mock inoculation and at 12h and 32h after inoculation (HAI), because these times represent stages of fungal penetration attempts and haustorium expansion, and both processes are influenced by RACB (Hoefle *et al.*, 2011). *PR* genes showed an enhanced expression level in supersusceptible *CA RACB* barley 12h after mock inoculation. Conversely, *PR* genes were underexpressed 12h after mock inoculation in less susceptible *RACB* RNAi barley when compared with the WT (Fig. 3A). At 32h, similar and partially stronger de-regulation of *PR* genes was observed in *RACB*-transgenic barley. However, this was not statistically significant for each individual *PR* gene (Fig. 3A). All four *PR* genes were up-regulated after inoculation with *Bgh* in the WT. At 12 and 32 HAI, *CA RACB* barley and *RACB* RNAi barley reacted similarly to the WT when inoculated with *Bgh* (Fig. 3B). Quantitative differences in the strength of the *PR* gene expression post-inoculation are explained by differences in constitutive gene expression. *CA RACB* barley reacted to *Bgh* with a less strong *PR* gene expression response because genes were already expressed at a higher level without inoculation. *JIP* gene expression was not strongly deregulated in *RACB*-transgenic barley. However, there was slightly enhanced expression of *JIP23* and *JIP60* in *CA RACB* barley (Fig. 3A, C). Other effects of genotype or inoculation on *JIP* gene expression were less consistent between the two sampling times. At 12 and 32 HAI with *Bgh*, the differences in gene expression between WT and *RACB*-transgenic genotypes were less pronounced when compared with the situation without inoculation (compare Fig. 3A and C). However, inoculated *CA RACB* barley still expressed single *PR* genes and JIP23 on an up to a 2.7-fold higher level than the WT, whereas *RACB* RNAi barley expressed the same genes at the level of the WT or below. Together, the *RACB* transgenes influenced defence gene expression but this did not reflect altered susceptibility of *RACB*-transgenic barley.

### RACB operates in root trichoblast polarity of barley

**Fig. 4.**
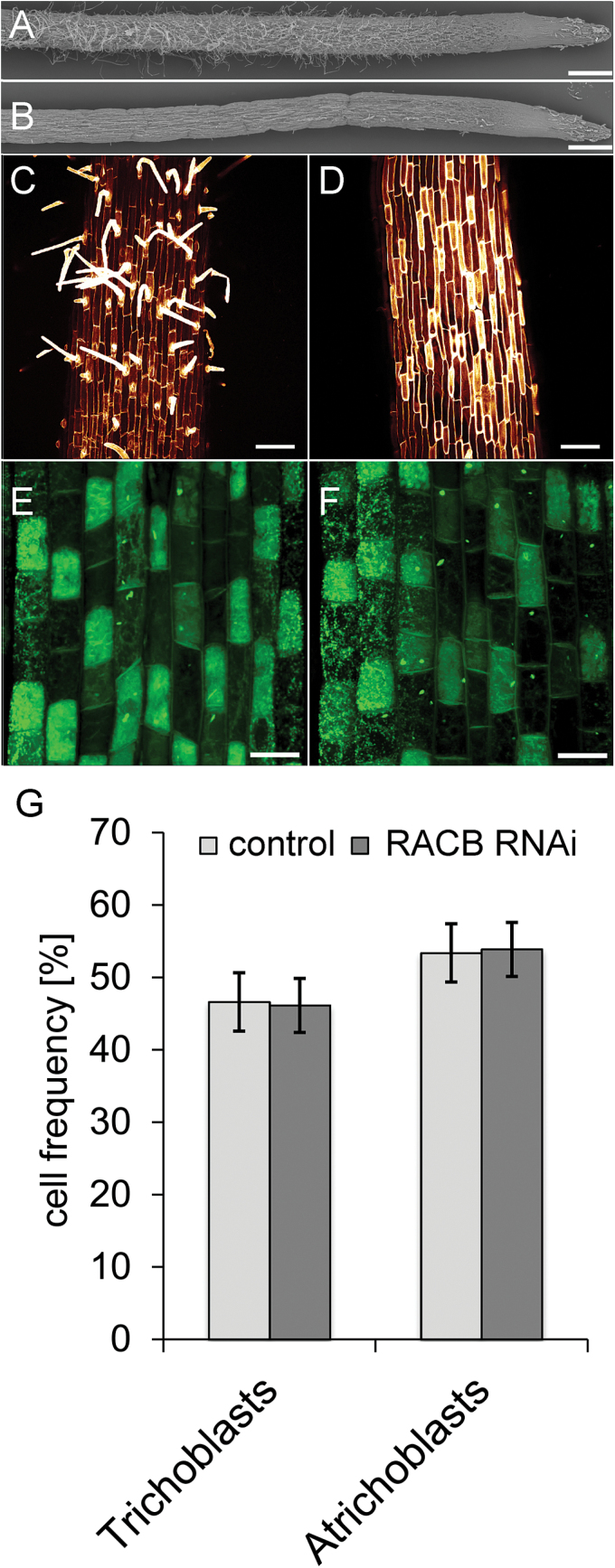
Root hair phenotype of *RACB* RNAi plants. (A) SEM of barley roots. Root hairs develop on azygous controls (non-transgenic segregants from RNAi plants). (B) *RACB* RNAi plants do not show root hair outgrowth. (C) Detailed view of the azygous control barley root stained with propidium iodide. Propidium iodide intensely stains root hairs. (D) Detailed view on a root segment of *RACB* RNAi plants, which corresponds to that of the control in Fig. 3C. (E) Rhodamin-123 staining of a root segment close to the tip of an azygous control root in which plasma-rich trichoblasts differentiate from more vacuolized atrichoblasts. (F) Rhodamin-123 staining of a root segment of *RACB* RNAi plants, which corresponds to that of the control in Fig. 3E. (G) Counting of trichoblasts reveals no differences in relative frequencies of cell types in azygous versus *RACB* RNAi barley roots. Columns show the mean of 30 root samples with a total of 1844 (control) or 1846 cells (*RACB* RNAi) counted. Error bars show the SD of the mean. Scale bars: A, B, 1mm; C, D, 100 µm; E, F, 25 µm. (This figure is available in colour at *JXB* online.

Because RACB did not regulate basal immune responses in a way that would explain its function as a susceptibility factor, we hypothesized that RACB’s role in plant cell development could support pathogenesis. We therefore studied *RACB* RNAi-mediated developmental failure in more detail. We first confirmed by SEM that knock down of *RACB* mediates inability to form root hairs (Fig. 4A, B). The roots showed a dramatic reduction of root hair outgrowth and, even if occasionally trichoblast protrusions were formed, they remained short. This has been similarly reported before for two independent transgenic *RACB* RNAi events (Hoefle *et al.*, 2011). Since barley root hairs can only develop from short epidermal trichoblasts, we compared the epidermal cell size pattern and number of trichoblasts in *RACB* RNAi plants and non-transgenic sister plants that lost the silencing cassette due to segregation (azygous control). Trichoblasts are shorter than atrichoblasts at late stages of root hair outgrowth (Marzec *et al.*, 2013). PI staining of comparable root sections of root hair initiation showed the occurrence of a typical pattern of shorter and longer cells in both *RACB* RNAi roots and the control (Fig. 4C, D). However, identification of short cells as trichoblasts was only possible after root hair outgrowth. Thus we used the fact that trichoblasts differ from atrichoblasts in size of vacuoles, density of cytoplasm, and number of cell organelles (Marzec *et al.*, 2013), and stained barley roots with the mitochondrial dye R123. Trichoblasts showed a more intense staining by R123 due to their higher number of mitochondria and denser cytoplasm compared with atrichoblasts. This allowed for the identification and quantification of trichoblasts before any obvious differences in cell expansion occurred. Qualitative and quantitative evaluation revealed that *RACB* RNAi plants were able to form trichoblasts in the same amount and pattern as azygous controls (Fig. 4E–G). Hence, *RACB* RNAi plants are able to specify root epidermal cells as trichoblasts but fail at bulging or tip growth at subsequent stages of root hair development (Schiefelbein, 2000).

### RACB is involved in leaf stomatal subsidiary cell formation

**Fig. 5.**
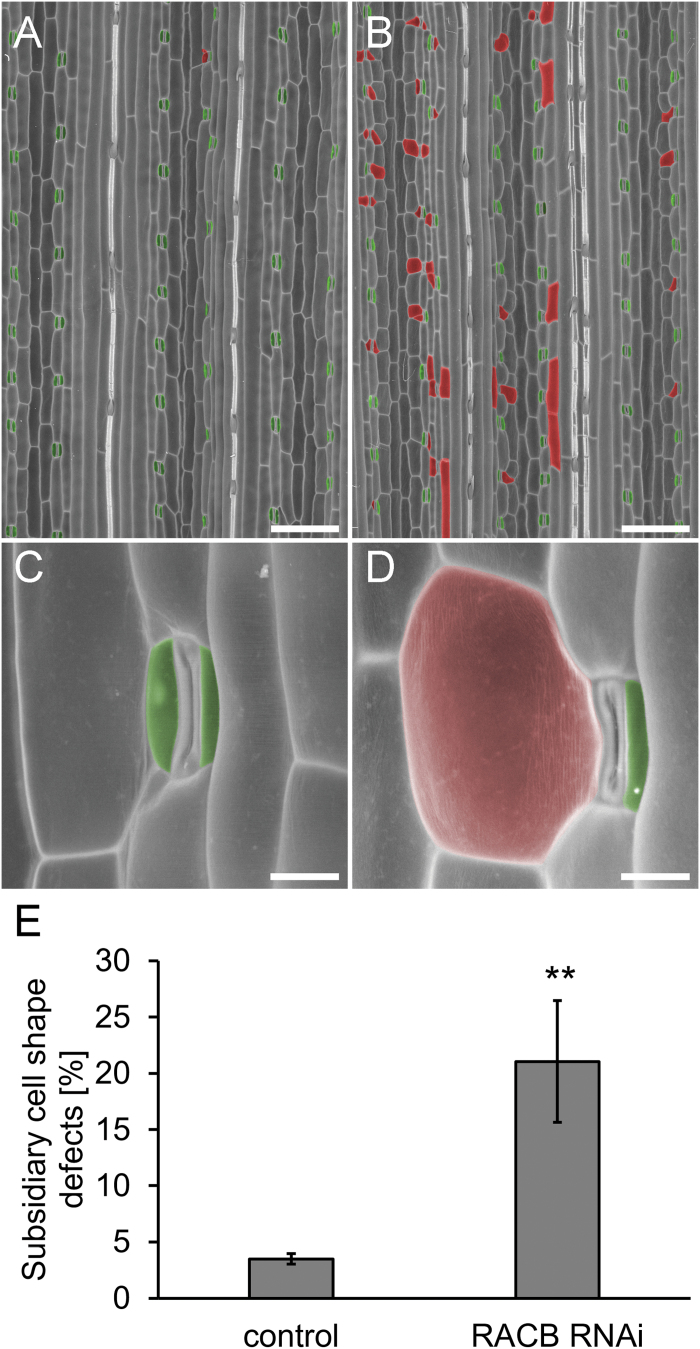
Stomatal subsidiary cell phenotypes of *RACB* RNAi plants. (A) Overview of barley second leaf epidermis by SEM. Stomata with subsidiary cells (green) develop properly on azygous controls. (B) *RACB* RNAi plants show frequent defects in formation of stomata subsidiary cells (red). (C) Detailed view of an azygous control barley leaf stoma. (D) Detailed view of a *RACB* RNAi barley leaf stoma. (E) Counting of stomatal defects reveals significant differences of frequencies in azygous control versus *RACB* RNAi barley roots. Columns show the mean of four leaf samples with 10 171 stomata counted on the controls and 9689 stomata counted on *RACB* RNAi leaves. Error bars show the SD of the mean (**; Student’s *t*-test *P*<0.01). Scale bars: A, B, 200 µm; C, D, 20 µm. A similar phenotype was observed in an independent transgenic *RACB* RNAi event 15/1-16 (Supplementary Fig. S1).

Type I ROPs are required for the asymmetric cell division of the subsidiary mother cell (SMC) resulting in the formation of a stomatal subsidiary cell and a pavement cell in *Zea mays* (Humphries *et al.*, 2011). We hence visualized patterns of epidermal cells in barley leaves using SEM. The subsidiary cells of *RACB* RNAi barley showed deformations of different degrees of severity or were often completely lacking. The SMC-derived pavement cells also exhibited serious defects in shape (Fig. 5B, D; Supplementary Fig. S1 at *JXB* online). The guard cells and other cell types of the leaf epidermis, however, were normally developed. We detected defective subsidiary cell formation on the leaf blade as well as on the leaf sheath, which develop from different meristems. Quantification of cell shape defects revealed that *RACB* RNAi barley failed to form normal stomatal subsidiary cells in ~20% of stomatal complexes whereas in azygous sister plants, this failure was observed in only 3.5% of stomata and was restricted to moderately distorted subsidiary and pavement cells in most cases (Fig. 5).

### RACB is involved in positioning of the nucleus in cells attacked by *Bgh*

**Fig. 6.**
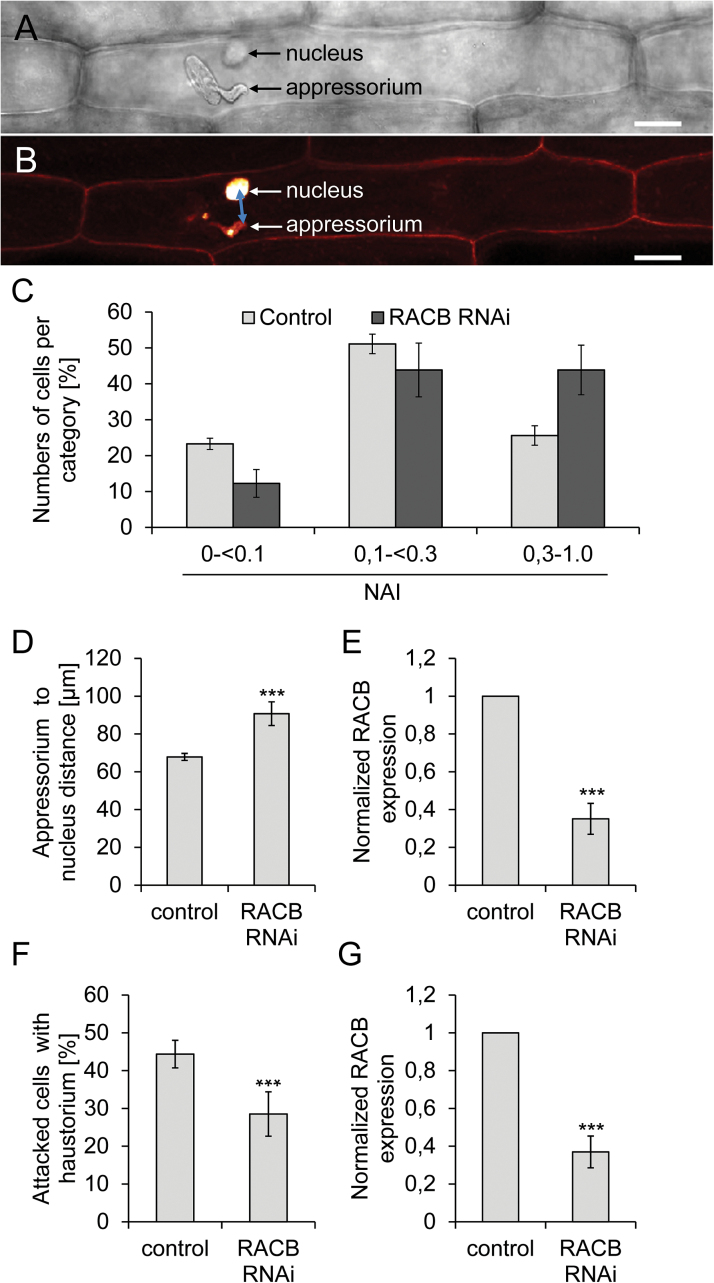
RACB influences positioning of the nucleus relative to the site of fungal attack. (A) Barley epidermal cell attacked by *Bgh* with the fungal appressorium and the plant nucleus (out of focus) visible in the transmission channel of the confocal laser scanning microscope. Size bar=25 µm. (B) The same barley epidermal cell as in (A) imaged by confocal laser scanning fluorescence microscopy. The fixed leaf was stained with propidium iodide to stain the fungus, the plant cell wall, and nucleus. The double-headed arrow indicates the distance from the site of fungal attack to the nucleus. Size bar=25 µm. (C) Considering that the nucleus is attracted by fungal attack, a nucleus attraction index (NAI) was calculated for 8h after inoculation (10 spores mm^−2^). First, the distance of the nucleus from the appressorium to the centre of the nucleus was calculated based on the horizontal distance (*x*/*y*-position, line in B) and the position of the nucleus in *z*. Subsequently, the NAI was calculated after normalizing to cell sizes (see the Materials and methods). The NAI was categorized into three groups representing the nucleus in close proximity of the fungus (0 to <0.1), in proximity to the fungus (0.1 to <0.3), and distant from the fungus (0.3 to <1). The NAI was measured on each of five or more azygous controls and *RACB* RNAi leaves at a minimum of 85 attacked cells per leaf. The χ^2^ test *P*-value for genotype-dependent differential distribution into the three NAI categories is *P*<0.001 for 8 HAI. A similar phenotype was observed in an independent transgenic *RACB RNAi* event 15/1-16 (Supplementary Fig. S1). (D) Absolute appressorium to nucleus distances in azygous controls and *RACB* RNAi plants at 8 HAI. (E) Expression level of *RACB* in leaf segments sampled in parallel to that used for the experiment in (D). (F) Frequencies of successful haustorium formation in azygous controls and *RACB* RNAi plants at 16 HAI. (G) Expression level of *RACB* in leaf segments sampled in parallel to that used for the experiment in (F). ***, Student′s *t*-test, *P*-value<0.001. Error bars show the SD of the mean of five individual leaves. (This figure is available in colour at *JXB* online.)

Positioning of the polarized nucleus is a common element of root hair outgrowth, subsidiary cell formation, and powdery mildew infection (Kita *et al.*, 1981; Opalski *et al.*, 2005; Humphries *et al.*, 2011; Griffis *et al.*, 2014). To analyse the influence of RACB on the position of nuclei in spatial association with fungal attack, we measured distances between nuclei and fungal appressoria in controls and *RACB* RNAi barley (Fig. 6A, B). To avoid mistakes due to cell shape effects, we focused on epidermal B-cell files between stomata and stomata-associated A-cell files (Koga *et al.*, 1990). Additionally, we normalized distances to the cell sizes to obtain an index (NAI, see material and methods) for each attacked cell that displays the distances in a cell size-independent manner. We chose 8 HAI as pre-penetration stage when appressoria are fully developed but haustoria are not yet established. At this point in time, nuclei were more distant from appressoria in *RACB* RNAi plants when compared with azygous control sister plants. This is evident by grouping the attacked cells by their individual NAIs (Fig. 6C). However, even without normalization to the cell sizes, the reduced attraction of the nucleus to the site of attempted penetration is apparent (Fig. 6D). To confirm that *RACB* RNAi plants are indeed less susceptible already at early stages of cellular interaction with *Bgh*, we scored frequencies of immature haustoria at 16 HAI, when haustoria reach a size that can be readily detected after staining with fluorescent wheat germ agglutinin (WGA). Similar to that observed previously for 48 HAI (Hoefle *et al.*, 2011), *RACB* RNAi plants allowed 35% less frequent establishment of haustoria when compared with azygous control sister plants (28.5% instead of 44.3% in the control) (Fig. 6F). We also confirmed that the *RACB* transcript amount was indeed reduced in the *RACB* RNAi plants when compared with azygous sister plants at 8 and 16 HAI. Therefore, we had cut the leaves at the mid rib and fixed one half of the leaf for determining the nucleus to appressorium distance or the haustorium frequency, respectively. The other halves of the leaves were used to measure *RACB* transcript abundance by RT–qPCR. *RACB* transcript abundance in *RACB* RNAi plants corresponded to 28% of the control level at 8 HAI and to 31% of the control level at 16 HAI (Fig. 6E, G). Together, the susceptibility factor *RACB* is involved in positioning of the nucleus in cells, which *Bgh* attempts to penetrate. In plants in which *RACB* expression is reduced by RNAi, the nucleus is more distant from the appressorium before the fungus penetrates and the fungus is subsequently less successful in penetration.

## Discussion

Susceptibility factors are plant components that serve the demands of a pathogen during disease development. However, the mode of action of susceptibility factors is often not well understood. They might be classified as negative regulators of host immunity or as host factors that support metabolic or developmental processes required for successful pathogenesis (Hückelhoven, 2005; Hückelhoven *et al.*, 2013; Lapin and Van den Ackerveken, 2013; van Schie and Takken, 2014). The latter, however, is challenging to provide evidence for, since it is difficult to distinguish whether failure of a pathogen to infect is because the host mutant does not properly support pathogenesis or, is due to an enhanced basal defence of that mutant. Our data support that RACB acts as a susceptibility factor through its function in cell polarity rather than by suppressing early MAMP-triggered immune responses or defence gene expression. Indeed, *CA RACB* enhanced *PR* gene expression in non-infected plants, and silencing of *RACB* lowered the level of *PR* gene expression. This is, however, not reflected in the resistance status of *RACB*-transgenic barley (Schultheiss *et al.*, 2005; Hoefle *et al.*, 2011) (Fig. 6) and therefore counterintuitive. Interestingly, expression of a dominant negative form of the Arabidopsis type I RAC/ROP protein DN ROP6 also causes enhanced defence gene expression. DN ROP6 expression further led to reduced penetration success and reduced reproductive success of the powdery mildew fungus *Golovinomyces orontii* in Arabidopsis. Genetic experiments with salicylic acid biosynthesis and signalling mutants suggested that defence gene expression can be uncoupled from powdery mildew resistance in this case (Poraty-Gavra *et al.*, 2013). Arabidopsis ROP6 might be involved in susceptibility to adapted powdery mildew independent of its function in salicylic acid signalling and defence gene expression. Together, perturbation of RAC/ROP signalling appears to alter plant defence gene expression but this cannot explain enhanced or reduced susceptibility of *RAC/ROP* mutants to powdery mildew.

Developmental host reprogramming is observed for mutualistic symbiosis such as root nodule development. However, there are few examples of pathogenic interaction with plant developmental programmes (Evangelisti *et al.*, 2014). The barley susceptibility factor RACB is involved in plant development and cytoskeleton organization (Opalski *et al.*, 2005; Pathuri *et al.*, 2008; Hoefle *et al.*, 2011). However, other RAC/ROP proteins are involved in regulating typical immune responses (Kawano *et al.*, 2014). We therefore studied typical early MAMP responses in *RACB*-misexpressing barley. This showed that barley reacts to MAMPs like other plants by early ROS production and MAPK activation. However, neither expression of CA *RACB* nor suppression of *RACB* expression greatly influences the ability of barley to respond quickly to fungal MAMP chitin or to the bacterial MAMP flg22. This suggests that RACB and the co-silenced RACD do not regulate canonical MAMP-triggered immunity in barley.

**Fig. 7.**
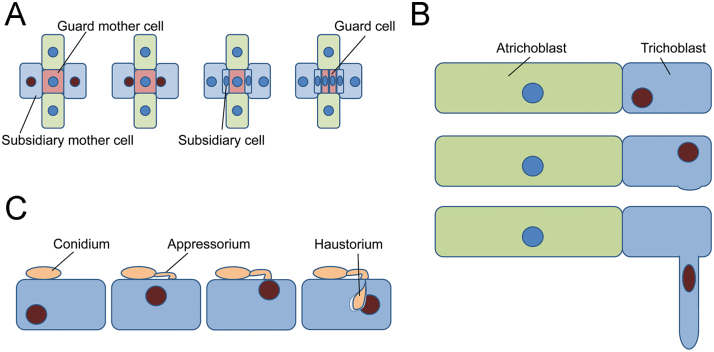
Positioning of the nucleus in polar epidermis cell development, which involves RACB in barley. (A) Subsidiary cell formation in stomata of grasses (Facette and Smith, 2012). Subsidiary cells develop from subsidiary mother cells (light blue) in that nuclei (dark-red) position to a guard mother cell. This allows for subsequent asymmetric cell division, in which a new cell wall is built between the subsidiary daughter cell and the adjacent epidermal daughter pavement cell (light blue). Subsequently guard cells develop from the guard mother cell by cell division. (B) Root hairs develop from short epidermal trichoblasts (blue). The nucleus moves to a position close to the future bulging site. The trichoblast bulges and growths out. Subsequently, the nucleus migrates into the tip-growing root hair and keeps a certain distance from the tip (Griffis *et al.*, 2014). Polar deposition of cell wall and membrane material is required for rapid growth of the hair. (C) The nucleus moves to the site of contact with a fungal appressorium and future ingrowth of the fungal haustorium. Polar deposition of membrane and cell wall (extrahaustorial membrane and matrix) is required for rapid accommodation of the fungal haustorium.

*RACB* RNAi effects on fungal success to develop haustoria can be observed in a cell-autonomous manner after transient induced gene silencing or after transient overexpression of *CA RACB* in single barley epidermal cells (Schultheiss *et al.*, 2002, 2003). This shows that RACB is required for fungal entry in a WT background, and reduced susceptibility of *RACB* RNAi barley is not a secondary consequence of developmental alterations. However, we considered developmental effects of *RACB* RNAi as instrumental to better understand the physiological role of *RACB* from which *Bgh* might profit. The involvement of RACB-like RAC/ROPs of dicots in development of root hairs and pollen tubes provoked the ‘inverted tip growth’ hypothesis (Schultheiss *et al.*, 2003). According to this, *Bgh* profits from RACB’s function in polar cell growth for inward growth of the fungal haustorium into an intact epidermal cell that surrounds the haustorium with a host-derived extrahaustorial membrane and matrix. Stable transgenic RNAi-mediated silencing of *RACB* provided first evidence for this hypothesis because a reduction of the *RACB* transcript level in *RACB* RNAi plants caused a dramatic reduction of frequency and size of hairs on the root epidermis and a strong reduction of frequency and size of *Bgh* haustoria in the leaf epidermis at 48 HAI (Hoefle *et al.*, 2011). In contrast, stable or transiently overexpressed *CA RACB* supports establishment of haustoria but induces isotropic instead of polar root hair growth (Schultheiss *et al.*, 2003; Pathuri *et al.*, 2008, 2009). In Arabidopsis, root hair development is dependent on ROP signalling. ROP proteins accumulate at the site of root hair initiation, and constitutively activated ROPs abolish root hair polarity whereas dominant negative ROPs restrict root hair development (Molendijk *et al.*, 2001; Jones *et al.*, 2002). The receptor-like kinase Feronia activates ROP2 for root hair development (Duan *et al.*, 2010). The positioning of root hairs is spatially controlled by the ROP-GDP dissociation inhibitor SCN1, and different ROP-GEFs influence the number, localization, and length of root hairs (Carol *et al.*, 2005; Huang *et al.*, 2013). The NADPH oxidase RBOHC is a potential ROP effector protein and required for root hair formation (Foreman *et al.*, 2003; Jones *et al.*, 2007). Also in barley a SCN1 homologue and ROS might function in root hair development (Kwasniewski *et al*., 2010, 2013). Actin microfilaments, microtubules, Ca^2+^ gradients, and ROS together appear to orchestrate polar root hair initiation and growth. Interestingly, all these components are influenced by ROP signalling in root hairs (Molendijk *et al.*, 2001; Jones *et al.*, 2002; Carol and Dolan, 2006; Yang *et al.*, 2007; Takeda *et al.*, 2008) and play a role in interactions of plants with powdery mildew fungi (Kobayashi *et al.*, 1997; Kim *et al.*, 2002; Hückelhoven and Kogel, 2003; Felle *et al.*, 2004; Hoefle *et al.*, 2011; Dörmann *et al.*, 2014). In the interaction of barley with *Bgh*, microfilament and microtubule organization are strongly influenced by RACB or by RACB-associated signalling components (Opalski *et al.*, 2005; Hoefle *et al.*, 2011; Huesmann *et al.*, 2012). In barley, only short plasma-rich epidermal cells, which gained identity as trichoblasts, are capable of initiating root hairs, whereas long expanding and highly vacuolized atrichoblasts remain hairless (Marzec *et al.*, 2013). We analysed cell identity of epidermis cells in the root differentiation zone and root transition zone of *RACB* RNAi barley (Verbelen *et al.*, 2006). This suggests that the knock down of *RACB* does not change trichoblast identity (Fig. 4E, G) but limits the ability of trichoblasts to undergo root hair initiation, bulging, and tip growth. Concerning the role of ROPs in root hair development (Molendijk *et al.*, 2001; Jones *et al.*, 2002; Singh *et al.*, 2008), this is probably caused by the inability to establish and maintain cell polarity in the trichoblasts, which is required for root hair development. Together, this supports that RACB acts in cell polarization during root hair initiation and tip growth. *Bgh* might profit from a similar function for RACB during initiation of and progressive ingrowth of the fungal haustorium into the leaf epidermis. Root hair formation always goes along with specific nucleus positioning in the trichoblasts throughout all phases of root hair development (Ketelaar *et al.*, 2002; Čiamporová *et al.*, 2003) (Fig. 7). Similar to this, the precise positioning of the nucleus of the SMC next to the guard mother cell (GMC) is the first visible indication for SMC polarization during stoma development in barley and maize. This SMC polarization is required for asymmetric cell division, resulting in a small-volume subsidiary cell and a large-volume epidermal pavement cell (Fig. 7) (Facette and Smith, 2012). The site-directed nucleus migration and polarization of the SMC is highly ROP regulated. It is thought that maize ROP2 and ROP9 stimulate the formation of a polar actin patch after their own accumulation at the anticlinal interface of the GMC. Maize *rop2/rop2;rop9*/+ mutants show a similar subsidiary cell formation defect to that which we examined on our barley *RACB* RNAi plants (Fig. 5) (Humphries *et al.*, 2011; Facette and Smith, 2012). Considering that ROP2 and ROP9 are extremely similar homologues of barley RACB (98% and 99% amino acid sequence identity, respectively), we suggest that RACB is involved in similar processes of SMC polarization and nuclear direction during subsidiary cell formation. The observed defects in subsidiary cell formation of *RACB* RNAi lines are best explained by a failure of asymmetric cell division. As a result of this, in most cases one cell, which originates from the undivided SMC (Fig. 5D), instead of two developed. In some other cases, the cell wall between the subsidiary cell and the SMC did not show the usual longitudinal orientation but was twisted, such that the cell wall of the subsidiary cell did not have a typically convex shape.

There is increasing awareness of an effector-triggered influence of microbes on plant development processes (Evangelisti *et al.*, 2014). Positioning of the nucleus is dynamic in both parasitic and mutualistic plant–microbe interactions. The formation of the pre-penetration apparatus in the response of legumes to hyphopodia formation by arbuscular mycorrhiza fungi involves attraction of the nucleus and its movement in front of the penetration hyphae (Genre and Bonfante, 2007). Therefore, we examined *RACB* RNAi plants for their capability for single cell polarization after fungal attack. We used positioning of the nucleus as a marker because it is common to root hair and subsidiary cell formation and to cell polarization in plant–microbe interactions (Fig. 7). The data support that nuclei closely associate with fungal appressoria in non-transgenic plants, as observed earlier (Kita *et al.*, 1981; Schmelzer, 2002; Opalski *et al.*, 2005). However, when we observed nuclei at 8 HAI before *Bgh* actually penetrated, nuclei appeared less attracted by fungal appressoria in *RACB* RNAi plants because NAIs were shifted to higher values and the absolute nucleus to appressorium distance was higher when compared with azygous controls (Fig. 6). This may indicate reduced single cell polarization of the attacked *RACB* RNAi cells at an early stage of plant–pathogen interaction and that RACB may be involved in positioning the nucleus in response to a fungal penetration attempt. The nucleus is confined and connected to the site of attack by both microtubules and microfilaments (Opalski *et al.*, 2005; Hoefle *et al.*, 2011). Since RAC/ROP proteins are key regulators of the plant cytoskeleton, and the cytoskeleton is an important target of plant pathogens (Cheong *et al.*, 2014; Porter and Day, 2015), it is logical that RACB mutants show nucleus positioning phenotypes in combination with an altered susceptibility. The role, however, of nucleus positioning during plant–pathogen interaction is hardly understood. Movement of the nucleus correlates with cytoplasmic aggregation at the sites of fungal attack and with subsequent secretion events for formation of cell wall appositions. In interaction with filamentous pathogens, cell polarization, and nuclear attraction is often less frequent and more transient in compatible interactions when compared with resistance (Schmelzer, 2002). Invasive hyphae of the Cowpea rust fungus are connected via host actin microfilaments and microtubules to the host plant nucleus in both compatible and incompatible interactions. However, nuclei are more often close to fungal hyphae in compatible interactions. The actin inhibitor cytochalasin E inhibits positioning of the nucleus close to fungal hyphae and hypersensitive cell death in resistant cultivars (Skalamera and Heath, 1998). Cytochalasin E also inhibits penetration resistance of barley to *Bgh* and *Erysiphe pisi* (Kobayashi *et al.*, 1997; Miklis *et al.*, 2007). Barley actin microfilaments and microtubules are strongly re-organized in cells that defend against fungal penetration (Kobayashi *et al.*, 1997; Opalski *et al.*, 2005; Hoefle *et al.*, 2011). Quantification of actin cytoskeleton patterns at 14–36 HAI suggested an association of cell polarity with penetration resistance to *Bgh* (Opalski *et al.*, 2005). In interaction with *Bgh*, stability of microtubules and polarization of both microfilaments and microtubules is influenced by RACB or by the RACB-interacting proteins MAGAP1 (MICROTUBULE-ASSOCIATED ROP GTPASE ACTIVATING PROTEIN 1) and RBK1 (ROP BINDING KINASE 1) (Opalski *et al.*, 2005; Hoefle *et al.*, 2011; Huesmann *et al.*, 2012). Together, this strongly suggests a function of polarity in penetration resistance. On the other hand, polar secretory events are also required for fungal accommodation in intact cells. Similar components of membrane transport act in penetration resistance and in formation of perimicrobial compartments in compatible plant–microbe interactions (Dörmann *et al.*, 2014). Additionally, the nucleus is a target of virulence effectors of diverse plant pathogens including powdery mildew (Wessling *et al.*, 2014). Cell polarization and movement of the nucleus may thus be important for basal penetration resistance. However, *Bgh* might also co-opt this during host cell re-programming for fungal accommodation. Additionally, *Bgh* might profit from a host cell developmental programme for polar growth including local cell wall remodelling and supply with sufficient building blocks for formation of the haustorial complex. As an obligate biotroph that has lost some essential gene functions during co-evolution with its host (Spanu *et al.*, 2010), *Bgh* might partially depend on support from its host. Our data support that RACB is a susceptibility factor that supports accommodation of fungal infection structures by its function in polar cell development.

## Supplementary data

Supplementary data are available at *JXB* online.

Figure S1. Polar cell development and the nucleus positioning phenotype of *RACB* RNAi event 15/1-16.

Supplementary Data
