# Supplementary material for: Barley disease susceptibility factor RACB acts in epidermal cell polarity and positioning of the nucleus
Source: J Exp Bot. 2016 Apr 7;67(11):3263–75. doi: 10.1093/jxb/erw141 (PMC4892720; doi:10.1093/jxb/erw141)
Supplement: Supplementary Data [file supp_67_11_3263__index.html]

Barley disease susceptibility factor RACB acts in epidermal cell polarity and positioning of the nucleus — Barley disease susceptibility factor RACB acts in epidermal cell polarity and positioning of the nucleus — Supplementary Data 

# Barley disease susceptibility factor RACB acts in epidermal cell polarity and positioning of the nucleus

## Supplementary Data

Data files

- supplementary\_figure\_S1.pdf - Supplementary Data
