# Supplementary material for: Barley disease susceptibility factor RACB acts in epidermal cell polarity and positioning of the nucleus
Source: J Exp Bot. 2016 Apr 7;67(11):3263–75. doi: 10.1093/jxb/erw141 (PMC4892720; doi:10.1093/jxb/erw141)
Supplement: Supplementary Data [file supp_erw141_supplementary_figure_S1.pdf]

Scheler et al. JXB, Supplementary Fig. 1

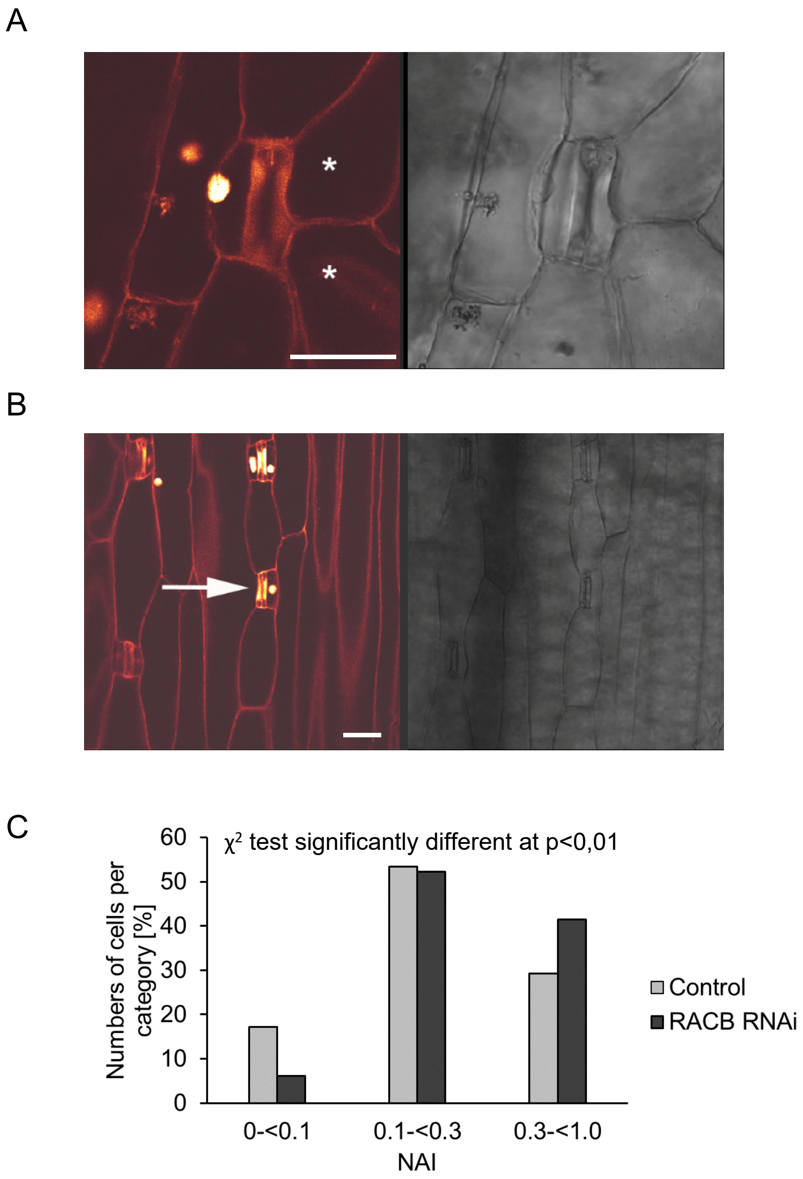

Fig. S1. Polar cell development and nucleus positioning phenotype of RACB RNAi event 15/1-16. (a,b) Stomatal subsidiary cell phenotype of *RACB* RNAi line 15/1-16. Barley second leaf epidermis shows defects (arrow, asterisks) in differentiation of subsidiary mother cells into normal subsidiary and pavement cells after PI staining. Size bars=50 µm. (c) Considering that the nucleus is attracted by fungal attack a nucleus attraction index (NAI) was calculated for 8 h after inoculation (10 spores mm^{-2}). First, the distance of the nucleus from the appressorium to the centre of the nucleus was calculated based on the horizontal distance and the nucleus position in z. Subsequently, the NAI was calculated after normalizing to cell sizes (see material and methods). The NAI was categorized in three groups representing the nucleus in close proximity of the fungus (0-<0,1), in proximity of the fungus (0,1-<0,3) and distant from the fungus (0,3-<1). NAI was measured on 65 azygous control and 58 *RACB* RNAi *Bgh* interation sites. χ^2 test p-values for genotype-dependent differential distribution into the three NAI categories is p<0,01.
